# Universal seeds for cDNA-to-genome comparison

**DOI:** 10.1186/1471-2105-9-36

**Published:** 2008-01-23

**Authors:** Leming Zhou, Jonathan Stanton, Liliana Florea

**Affiliations:** 1 Department of Computer Science, George Washington University, Washington DC, 20052, USA; 2 Department of Biochemistry and Molecular Biology, George Washington University, Washington DC, 20052, USA

## Abstract

**Background:**

To meet the needs of gene annotation for newly sequenced organisms, optimized spaced seeds can be implemented into cross-species sequence alignment programs to accurately align gene sequences to the genome of a related species. So far, seed performance has been tested for comparisons between closely related species, such as human and mouse, or on simulated data. As the number and variety of genomes increases, it becomes desirable to identify a small set of *universal *seeds that perform optimally or near-optimally on a large range of comparisons.

**Results:**

Using statistical regression methods, we investigate the sensitivity of seeds, in particular good seeds, between four cDNA-to-genome comparisons at different evolutionary distances (human-dog, human-mouse, human-chicken and human-zebrafish), and identify classes of comparisons that show similar seed behavior and therefore can employ the same seed. In addition, we find that with high confidence good seeds for more distant comparisons perform well on closer comparisons, within 98–99% of the optimal seeds, and thus represent universal good seeds.

**Conclusion:**

We show for the first time that optimal and near-optimal seeds for distant species-to-species comparisons are more generally applicable to a wide range of comparisons. This finding will be instrumental in developing practical and user-friendly cDNA-to-genome alignment applications, to aid in the annotation of new model organisms.

## Background

The next few years are expected to bring a significant increase in the number of available genomes, driven by advances in sequencing technologies [1]. As genome sequencing projects outpace the generation of native mRNA and protein sequences, gene annotation projects for these genomes will need to rely instead on cDNA information from other species. While existing alignment programs align cDNA and the corresponding genomic sequences accurately, they are inadequate for cross-species comparisons [2]. Beginning with blast [3,4], most alignment programs have used a *seed-and-extend *technique to produce local alignments, starting from exact or near-exact word matches *(seeds) *between the two sequences and extending them to a local alignment in several stages. Blast uses an exact match of 11 contiguous positions, represented by a vector of 1s (11_*c *_= 11111111111). Such a seed is called *continuous*. More recently, *spaced seeds *have been introduced, which allow wildcard positions in the seed pattern, marked with 0s. For instance, Kent and Zahler [5] used a seed that allowed for mismatches at the wobble codon position (WABA_12 _= 11011011011011011) to increase the sensitivity of cDNA-genomic sequence alignment. For the same *weight*, or number of 1 positions, spaced seeds were shown to perform better than continuous seeds in most cases [6].

Recently, Ma *et al*. [7] introduced a framework for predicting the sensitivity of a spaced seed given a model of alignment, and showed how to determine the theoretical seed sensitivity with an efficient dynamic programming method [8]. Given a Bernoulli model of alignments with the parameter *p *(*i.e*., the probability that any one alignment position is a match) estimated from the average sequence identity, they determined optimal seeds for human-mouse genomic sequence alignment. These seeds were later implemented in the programs PatternHunter and blastz [9]. Subsequent studies used increasingly complex alignment models, such as the order 1 inhomogeneous 3-periodic Markov models in [6,10] or the higher order (3–5) inhomogeneous Markov models of [11], often in the specialized context of coding sequences. Extensions of the seed models were proposed to further improve sensitivity or extend the range of applicability to non-nucleotide sequences, for instance multiple spaced seeds [12], or vector seeds [13]. In the latter, each position in the seed pattern is a real value representing the weight of a match or substitution at that position in the total match score, and a seed match is declared when the total score exceeds an a priori fixed threshold. However, these methods increase the memory and running time of searches, both of which are critical for practical high-throughput applications.

While early alignment and seed models used the traditional {0, 1} alphabet, to further improve the seed sensitivity transition-only wildcards were introduced to differentiate between transitions and transversions, first implemented in blastz [9]. Later, Noé and Kucherov [14] formalized the concept and showed that spaced seeds with transitions extend the sensitivity range of {0, 1} seeds, and Zhou and Florea [11] additionally provided a framework for specificity calculation, and showed that they offer better sensitivity-specificity tradeoffs than {0, 1} seeds in practice.

When comparing coding sequences, such as in cDNA-to-genome alignments, the codon organization, higher-order position dependencies [15] and specific transition-transversion biases [16] inherent in the gene sequences are likely to be reflected in the alignment patterns. In [6,10], a three-state Markov model of order 1 is used to simulate the three codon positions, while Brejová *et al*. [17] introduce two models, the first a three-state Markov model of order 0, and the second a more complex Bernoulli formulation in which each codon is modeled independently. Recently, we proposed to use higher order (3–5) inhomogeneous Markov models with transitions [11] to capture both transition-transversion biases and sequence compositional patterns.

As the number of sequenced organisms increases, the range of possible pairwise species comparisons will grow quadratically with the number of species. For practical reasons it becomes desirable to identify a small number of seeds that would perform well for a wide range of comparisons, at varying evolutionary distances. Earlier, Choi *et al*. [18-20] determined and analyzed best seeds for genomic sequence comparisons for several sequence identity levels under a Bernoulli model of alignment, in a greatly simplified model of evolutionary distance. At low weights (10–12), seeds performed optimally or near-optimally across a wide range of sequence similarity levels. At higher weights, however, there was significant fluctuation in seed sensitivity, which led them to the hypothesis that different seeds will be needed for each type of comparison. This simple *p*-level representation of evolutionary sequence divergence is likely inadequate for coding sequences, which are under a more diverse set of evolutionary pressures. We approach the question of designing *universal *good seeds for cDNA-to-genome comparisons, *i.e*. seeds that perform well for a large number of comparisons, starting from a complex representation of alignment as a high order 3-periodic inhomogeneous Markov model incorporating transitions [11]. Using comparisons between human and four others species (mouse, dog, chicken and zebrafish) to sample a wide range of evolutionary distances, we analyze the distributions of seed sensitivities statistically to characterize and identify universal good seeds. In the remainder of this section, we provide a brief introduction to the problem of designing optimal spaced seeds.

### Spaced Seeds

#### {0,1} spaced seeds

Alignment programs typically use short exact or approximate matches of an *a priori *specified pattern *(seed) *to detect local alignments between two sequences. A *continuous *seed, such as the one used in blast [3,4], requires an exact match of a fixed length *k *between the sequences, and is represented as a vector of 1s (11_*c *_= 11111111111). In contrast, spaced seeds [7] allow for approximate matches by including wildcard positions in the pattern, marked with 0s (*e.g*., WABA_12 _= 11011011011011011). The number of 1 positions in the pattern is called the *weight *of the seed, and the length of the pattern is called the *span*. Conventionally, seeds must start with a 1 position. In keeping with previous studies, we will use a fixed seed span *k *= 22 [6,10,11].

An alignment is represented as a string of 0s (mismatches) and 1s (matches) generated from a model M, for instance a Bernoulli or a Markov model. A seed *S *= *s*_1 _... *s*_*k*_, *s*_1 _= 1, is said to detect the alignment *w *= *w*_1 _... *w*_*L *_∈ {0, 1}^*L *^if there is an approximate match for the pattern in the alignment string such that all 1 positions in the seed pattern map to 1 positions in the alignment, *i.e*. there exists *i *= 1..*L *- *k *+ 1 such that *w*_*i*+*l*-1 _= 1 for all *l *with *s*_*l *_= 1. If such an *i *exists, the seed is said to occur in the alignment *w *at position *i*. The theoretical sensitivity of a seed is then defined as the probability that it will detect a random alignment of length *L *generated from the model M, or equivalently: *Sn*(*S*) = *P*({*w ∈ *{0, 1}^*L*^|*S *detects *w*}) [6,8]. Traditionally, the alignment length *L *is 64, determined as the average length of a gap-free alignment in human-mouse comparisons. By definition, an *optimal seed *is a seed with the highest sensitivity. For a given seed, its sensitivity can be computed exactly using dynamic programming [8,10,21]. Optimal seeds can then be produced by exhaustively searching the seed space [8], while close approximations can be obtained with fast heuristics, such as hill-climbing [6,10] or exploiting the seed structure to reduce the search space [20].

#### {0, 1, x} spaced seeds for cDNA-to-genome alignment

Unlike genomic sequences, gene sequences exhibit higher order dependencies between positions [15], more pronounced transition-transversion biases [16], and distinct manifestations at the three codon positions, which are likely to translate into alignment patterns. To account for these characteristics, in previous work [11] we proposed a framework that differentiated between transitions and transversions, by using an additional alphabet symbol *x*, as well as among the three codon positions, using a third order inhomogeneous 3-periodic Markov model of cDNA-to-genome alignments. In the seed pattern, *x *marks a position that allows transitions but not transversions, while in the alignment model it simply represents a transition. The weight of the new symbol is 0.5. We denote (*n*_1_, *n*_0_, *n*_*x*_) the class of seed patterns with *n*_1_, *n*_0 _and *n*_*x *_symbols of 1, 0 and *x*, respectively, *n*_1 _+ *n*_0 _+ *n*_*x *_= *k*. For a given weight *W*, there may be multiple (*n*_1_, *n*_0_, *n*_*x*_) combinations with *n*_1 _+ 0.5·*n*_*x *_= *W *. For instance, for the weight *W *= 10 and span *k *= 22, the following combinations are possible: (10, 12, 0), (9, 11, 2), ..., (0, 2, 20).

In the following sections, we investigate the behavior of seeds, and in particular best seeds, among four comparisons or cDNA-to-genome alignment models (human-mouse, human-dog, human-chicken and human-zebrafish) to obtain a robust characterization of universal good seeds.

## Results

The repertoire of species to be sequenced is expected to increase dramatically over the next few years, driven by new, more effective and increasingly reliable sequencing technologies. As the number and phylogenetic diversity of genomes increases, designing optimized seeds for each pair of compared species quickly becomes impractical. It becomes desirable to identify a limited number of seeds that perform well, if not optimally, for a large number of comparisons. We call these *universal *good seeds. We examine seeds for four comparisons between species with significantly different evolutionary distances and mutation patterns (human-mouse, human-dog, human-chicken and human-zebrafish). For simplicity, we will refer to each comparison by the name of the second organism (DOG, MUS, CHK, ZFS). By analyzing the distribution of seed sensitivities between models statistically, we identify strategies for designing universal good seeds.

### Universal good seeds

We address two questions: First, are there groups of comparisons, or equivalently alignment models, that produce similar behavior of all seeds? We call such models *seed-equivalent*, and one optimal seed would then satisfy all comparisons in a seed-equivalence group. Second, are there *universal *good seeds, *i.e*. seeds that are optimal or near-optimal for a large range of comparisons and evolutionary distances?

**Figure 1:**
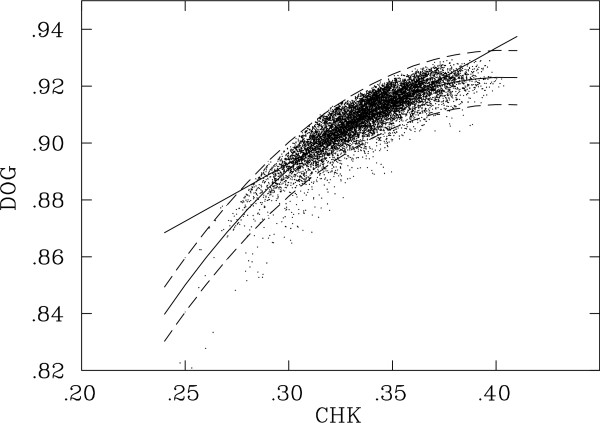
Scatterplot of seed sensitivity values between the CHK and DOG comparisons, for weight *W *= 16 and for the (16, 6, 0) combination. The linear and non-linear regression (solid) and the 95% confidence interval (dotted) curves are shown.

To answer these questions, we started by calculating seed sensitivities exhaustively in the four models for three weights, *W *= 12, 14, 16, for the (12, 10, 0), (14, 8, 0) and (16, 6, 0) combinations. We then compared the distributions of values between any two models using statistical regression methods to determine seed trends, by measuring the 95% confidence interval for a seed when projected via the regression curve, and to identify outliers, or seeds that have significantly different behavior (at > 2.5*σ *from the regression curve) between the two models. Although linear and non-linear regression models showed similar goodness of fit, an order 3 non-linear method was chosen because it provided a more conservative estimate of predicted values among the top scoring seeds, which are particularly relevant to this study. The scatter plot of seed values between the CHK and DOG distributions and the two regression curves, linear and non-linear, produced with the R statistical analysis package [22] are shown in Figure 1. Scatterplots for all pairwise comparisons are shown in [Additional file 1].

#### Determining seed-equivalent models

For most pairs of models, the 95% confidence interval for the predicted seed values (2·*t*_*α*/2_*σ*_*y *_in Table 1) is less than or close to 0.01, meaning that with high probability the observed seed sensitivity in the second model is within close vicinity ($\hat{y}$ - *t*_*α*/2_*σ*_*y*_, $\hat{y}$ + *t*_*α*/2_*σ*_*y*_) of the predicted value, $\hat{y}$. When seed values can be so closely predicted between both (*X*, *Y*) and (*Y*, *X*), the two models *X *and *Y *can be deemed seed-equivalent. In our case, DOG and MUS, and CHK and ZFS, respectively, are seed-equivalent despite the difference in their sensitivity ranges. Interestingly, seed values appear to also be accurately projected from a reference model *X *describing a more distant evolutionary relationship, to a compared model *Y *representing a closer comparison, but not conversely. We explore this observation below.

**Table 1:** Comparison of seed sensitivity distributions between models. Seed population sizes for (12,10,0), (14,8,0), (16,6,0) are 352716, 203490 and 54264, respectively. Explanation of columns: *y*_*min*, *max *_– minimum and maximum sensitivity in the compared model; *x*_*max *_– maximum sensitivity in the reference model; *t*_*α*/2_*σ*_*y *_– half the length of the 95% prediction interval (*t*_*α*/2 _= 1.96 when *α *= 0.05); *T*(*x, y*) = ((*a *+ *bx*_max _+ $cx_{\max}^{2}$ + $dx_{\max}^{3}$) - *t*_*α*/2_*σ*_*y*_)*/y*_max_, where *a*, *b*, *c *and *d *are the coefficients of the regression curve, and *σ*_*y *_is the estimated regression standard error of prediction for a given *x *value. Because the number of values is large, *σ*_*y *_≃ *σ *for all *y*. Outliers are determined as those points that satisfy either of the following two criteria: *y *- $\hat{y}$ > 2.5*σ *(U) or $\hat{y}$ - *y *> 2.5*σ *(L). Here, $\hat{y}$ = *a *+ *bx *+ *cx*^2 ^+ *dx*^3^.

	*x*_*max*_	*y*_*max*_	*y*_*min*_	*t*_*α*/2_*σ*_*y*_	*T*(*x, y*)	*U*	*L*
W = 12							

CHK-DOG	0.742	0.992	0.936	0.002	0.996	0.04%	1.75%
DOG-CHK	0.992	0.742	0.429	0.028	0.947	0.99%	0.09%
CHK-MUS	0.742	0.964	0.827	0.005	0.991	0.01%	1.74%
MUS-CHK	0.964	0.742	0.429	0.017	0.978	1.70%	0.00%
CHK-ZFS	0.742	0.476	0.196	0.006	0.995	1.52%	0.10%
ZFS-CHK	0.476	0.742	0.429	0.006	0.984	0.04%	1.36%
DOG-MUS	0.992	0.964	0.827	0.004	0.996	1.63%	0.03%
MUS-DOG	0.964	0.992	0.936	0.001	0.999	0.02%	1.95%
DOG-ZFS	0.992	0.476	0.196	0.033	0.866	1.59%	0.01%
ZFS-DOG	0.476	0.992	0.936	0.003	0.995	0.03%	1.71%
MUS-ZFS	0.964	0.476	0.196	0.023	0.934	1.98%	0.00%
ZFS-MUS	0.476	0.964	0.827	0.006	0.988	0.01%	1.70%

W = 14							

CHK-DOG	0.567	0.971	0.900	0.005	0.990	0.00%	1.71%
DOG-CHK	0.971	0.567	0.335	0.027	0.913	3.26%	0.00%
CHK-MUS	0.567	0.901	0.758	0.009	0.985	0.00%	1.66%
MUS-CHK	0.901	0.567	0.335	0.018	0.953	1.89%	0.00%
CHK-ZFS	0.567	0.299	0.132	0.005	0.979	1.61%	0.01%
ZFS-CHK	0.299	0.567	0.335	0.007	0.983	0.00%	1.48%
DOG-MUS	0.971	0.901	0.758	0.005	0.996	1.43%	0.01%
MUS-DOG	0.901	0.971	0.900	0.002	0.997	0.01%	1.69%
DOG-ZFS	0.971	0.299	0.132	0.024	0.836	1.65%	0.00%
ZFS-DOG	0.299	0.971	0.900	0.006	0.988	0.00%	1.66%
MUS-ZFS	0.901	0.299	0.132	0.018	0.882	2.09%	0.00%
ZFS-MUS	0.299	0.901	0.758	0.012	0.978	0.00%	1.70%

W = 16							

CHK-DOG	0.405	0.930	0.821	0.010	0.982	0.00%	1.98%
DOG-CHK	0.930	0.405	0.248	0.021	0.907	1.65%	0.00%
CHK-MUS	0.405	0.808	0.633	0.013	0.976	0.00%	1.98%
MUS-CHK	0.808	0.405	0.248	0.015	0.944	1.86%	0.00%
CHK-ZFS	0.405	0.176	0.086	0.004	0.959	1.99%	0.00%
ZFS-CHK	0.176	0.405	0.248	0.006	0.986	0.00%	1.90%
DOG-MUS	0.930	0.808	0.633	0.006	0.991	1.62%	0.00%
MUS-DOG	0.808	0.930	0.821	0.003	0.996	0.04%	1.49%
DOG-ZFS	0.930	0.176	0.086	0.015	0.804	1.68%	0.00%
ZFS-DOG	0.176	0.930	0.821	0.012	0.976	0.00%	1.97%
MUS-ZFS	0.808	0.176	0.086	0.012	0.858	1.96%	0.00%
ZFS-MUS	0.176	0.808	0.633	0.018	0.964	0.00%	1.97%

#### Determining universal seeds

With the same argument as above, when the margin of error *t*_*α*/2_*σ*_*y *_is small, with high confidence high-scoring seeds in the reference model are expected to lie at the top of the sensitivity range in the compared model. Hence, good seeds will largely be shared between the models. To measure how closely the predicted value of the optimal seed in the reference model (*x*_*max*_) approaches the optimal (real) seed in the compared model (*y*_*max*_), we used the statistical lower bound of the predicted interval for *x*_*max*_, as a worst case scenario, and compared it with *y*_*max*_. These ratios are shown as *T *(*x, y*) in Table 1. As expected, the values are consistently above 0.98 between the seed-equivalent models, but also when a seed in a more distant model is projected onto a closer model, and the trends are more pronounced with the larger seed weights. This level of accuracy in predicting near-optimal seeds by regression projection, albeit statistical, is comparable with that obtained with the heuristic algorithm proposed in [20]. To rule out the effects of outliers among the top-scoring candidates, we identified those seeds that fall significantly outside of the 95% confidence range (Table 1, columns 7 and 8). Although roughly 1–2% of seeds are expected to place outside the predicted range, they are scattered along the sensitivity range for the model.

Collectively, these findings suggest that simply selecting top scoring seeds in the most distant model, in our case ZFS, would lead to good seeds for all other models, and therefore gives a simple strategy for determining universal good seeds.

### Evaluation

**Figure 2:**
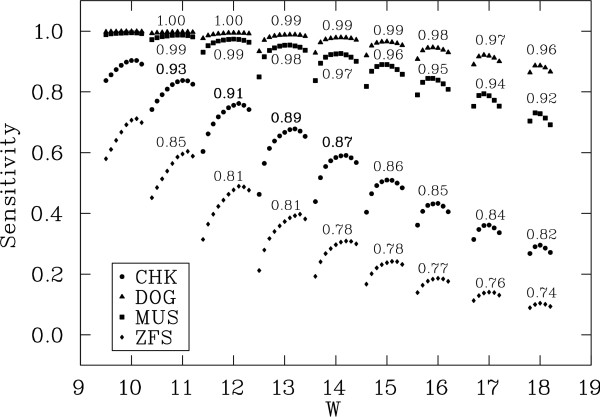
Sensitivity maxima for DOG, MUS, CHK and ZFS comparisons, for seeds of weight *W *= 10..18. For each weight *W*, (*n*_1_, *n*_0_, *n*_*x*_) combinations are shown right-to-left starting with *n*_*x *_= 0 and subsequently increasing *n*_*x*_. Sensitivity drop rates between consecutive weights are shown at the top of the plots.

To probe the universality of seeds, we evaluate seeds optimized for one comparison on the three others. For each comparison, we determine best seeds for most weights of practical interest, *W *= 10..16, for all (*n*_1_, *n*_0_, *n*_*x*_) combinations. Table 2 lists the optimal seed under our fixed span model (*k *= 22) for each weight for the four comparisons, and the complete list including all combinations is in [Additional file 2]. Figure 2 shows the sensitivity maxima that can be achieved by seeds for each combination. For each comparison (DOG, MUS, CHK, ZFS) the maximum overall sensitivity declines steadily as the seed weight increases, at a rate roughly proportional to the percentage of matches in the alignment (*p*_*d *_= 89.0%, *p*_*m *_= 85.0%, *p*_*c *_= 75.5% and *p*_*z *_= 68.7%). Within each weight group, the sensitivity maxima vary, sometimes dramatically, between (*n*_1_, *n*_0_, *n*_*x*_) combinations: seeds with the largest number of transitions consistently score the lowest, and seeds with a small number of transitions, depending on the species (2–4 for CHK and ZFS, 6–8 for MUS and DOG), perform the best. We hypothesize that the optimal number of seed transitions for each comparison is determined by the transition-transversion ratio (*κ*) in the alignment model, and that more transition positions are expected in the optimal seeds as the ratio increases. Thus, DOG (*κ*_*d *_= 1.97) and MUS (*κ*_*m *_= 1.73) are the most likely to see the effects of transitions, while the effects on CHK (*κ*_*c *_= 1.17) and ZFS (*κ*_*z *_= 0.95) will be smaller. This ratio also determines the gain in sensitivity that can be obtained by seeds incorporating transitions compared to {0,1} seeds.

**Table 2:** Seeds optimized for the CHK, DOG, MUS, ZFS comparisons, for weight W = 10..16, using hill-climbing. For large weights (*e.g*., W ≥ 16), the fixed span *k *= 22 may significantly constrain the range of seeds, and therefore the seeds produced under this model may not be optimal in practice.

**Comparison**	*n*_1_	*n*_0_	*n*_*x*_	*W*	**Sensitivity**	**Seed**
CHK	9	11	2	10	0.9033819146	1x11011011x11000000000
CHK	9	9	4	11	0.8373594847	1xx1011011011xx1000000
CHK	10	8	4	12	0.7617553847	1xx1011011011xx1100000
CHK	11	7	4	13	0.6781533749	11x1101101x011xx110000
CHK	12	6	4	14	0.5907201266	11x11011011011xxx11000
CHK	12	4	6	15	0.5096393921	11x110xxx1011011011xx1
CHK	14	4	4	16	0.4328248093	11x110xx11011011011x11

DOG	8	10	4	10	0.9991951771	11xx1101x011x100000000
DOG	9	9	4	11	0.9976354479	11x110110x0x1x11000000
DOG	10	8	4	12	0.9943213958	11x110x1011x1x11000000
DOG	10	6	6	13	0.9882916262	11x1101x1x0xx011x11000
DOG	11	5	6	14	0.9790689537	11xx11011x0x0x1011x110
DOG	12	4	6	15	0.9652197277	11x110x1x010x1011x1x11
DOG	13	3	6	16	0.9461146848	11x110x1x01xx1011x1111

MUS	8	10	4	10	0.9946391036	1xx1011011xx1100000000
MUS	9	9	4	11	0.9868058410	11x1101x011xx110000000
MUS	9	7	6	12	0.9737371393	1x1101x010x1x011xx1000
MUS	10	6	6	13	0.9538071885	1x1101xx10x1x011x11000
MUS	11	5	6	14	0.9260863280	11xx110x1x010x1011x110
MUS	11	3	8	15	0.8901430490	11x110x1x01xx1011x1xx1
MUS	13	3	6	16	0.8445827211	11x1101xxx011x11011x11

ZFS	9	11	2	10	0.7113928043	1x11011011x11000000000
ZFS	10	10	2	11	0.6041894577	11x11011011x1100000000
ZFS	10	8	4	12	0.4894026703	x1x11011011011xx100000
ZFS	12	8	2	13	0.3972876500	11x11011011011x1100000
ZFS	12	6	4	14	0.3090795416	1xx110110x1011011x1100
ZFS	13	5	4	15	0.2419994803	11x1101101xx11011x1100
ZFS	14	4	4	16	0.1863098023	11xx11011011x11011x110

To validate the universal seeds, we measured the performance of seeds optimized for one comparison on each of the other three, both theoretically and on real data. For empirical evaluations, we tested the ability of each seed to detect orthologous exons between human and each of the other species. Table 3 lists the theoretical (T) and empirical (E) seed sensitivity values, calculated as described in Methods, averaged over each weight group. Theoretical values show very similar sensitivities for all four groups of seeds on all four comparisons. Empirical values are somewhat more varied, but they also indicate that all seeds perform optimally or near-optimally for multiple comparisons. In all cases, seeds designed for the more distant comparisons have near optimal performance for the closer ones. In particular, ZFS and CHK optimal seeds are widely applicable, and thus are good candidates for universal good seeds.

**Table 3:** Theoretical (T) and empirical (E) sensitivities of optimal seeds in the four models. Letters C, D, M, Z indicate the CHK, DOG, MUS and ZFS comparisons, respectively. C_*do *_represents sensitivity values for the optimal DOG seeds (*do*) when applied to the CHK (C) model. Values are averaged within each weight group.

**W**	**C**_*co*_	**C**_*do*_	**C**_*mo*_	**C**_*zo*_	**D**_*co*_	**D**_*do*_	**D**_*mo*_	**D**_*zo*_
10^*T*^	0.881	0.875	0.878	0.881	0.998	0.999	0.999	0.998
10^*E*^	0.904	0.896	0.897	0.905	0.983	0.984	0.984	0.982
11^*T*^	0.808	0.800	0.803	0.808	0.996	0.996	0.996	0.996
11^*E*^	0.876	0.859	0.856	0.875	0.976	0.978	0.976	0.976
12^*T*^	0.717	0.708	0.715	0.716	0.990	0.990	0.990	0.989
12^*E*^	0.822	0.819	0.822	0.831	0.967	0.970	0.968	0.967
13^*T*^	0.628	0.619	0.624	0.627	0.978	0.979	0.979	0.977
13^*E*^	0.779	0.756	0.761	0.790	0.954	0.954	0.953	0.953
14^*T*^	0.555	0.542	0.549	0.554	0.967	0.969	0.969	0.965
14^*E*^	0.735	0.706	0.731	0.763	0.943	0.942	0.943	0.943
15^*T*^	0.483	0.472	0.480	0.481	0.952	0.955	0.954	0.949
15^*E*^	0.706	0.676	0.686	0.733	0.927	0.927	0.926	0.930
16^*T*^	0.412	0.400	0.407	0.410	0.931	0.935	0.935	0.927
16^*E*^	0.667	0.610	0.627	0.679	0.912	0.909	0.910	0.910

**W**	**M**_*co*_	**M**_*do*_	**M**_*mo*_	**M**_*zo*_	**Z**_*co*_	**Z**_*do*_	**Z**_*mo*_	**Z**_*zo*_

10^*T*^	0.992	0.992	0.993	0.992	0.663	0.647	0.654	0.663
10^*E*^	0.978	0.978	0.979	0.978	0.745	0.716	0.725	0.751
11^*T*^	0.981	0.982	0.982	0.981	0.547	0.529	0.534	0.547
11^*E*^	0.970	0.969	0.969	0.969	0.678	0.636	0.626	0.676
12^*T*^	0.963	0.963	0.963	0.961	0.432	0.417	0.426	0.433
12^*E*^	0.957	0.958	0.958	0.957	0.570	0.562	0.565	0.591
13^*T*^	0.932	0.933	0.934	0.930	0.342	0.329	0.335	0.344
13^*E*^	0.940	0.938	0.940	0.941	0.504	0.464	0.474	0.525
14^*T*^	0.904	0.905	0.906	0.900	0.276	0.260	0.267	0.278
14^*E*^	0.925	0.922	0.927	0.929	0.436	0.394	0.425	0.488
15^*T*^	0.867	0.870	0.871	0.861	0.220	0.207	0.215	0.222
15^*E*^	0.909	0.906	0.908	0.913	0.395	0.358	0.365	0.447
16^*T*^	0.822	0.825	0.826	0.815	0.172	0.160	0.166	0.173
16^*E*^	0.889	0.882	0.886	0.890	0.350	0.283	0.302	0.368

## Discussion

While significant effort has gone in designing optimal seeds for comparing human and mouse sequences, a remarkably few other comparisons received any attention at all. With more species being sequenced over the next few years, identifying a small number of seeds that would perform optimally or near-optimally for a large range of comparisons is becoming essential. For genomic sequences, the sequence identity level has been used as a simple measure of evolutionary distance. Since the sequence composition of genes is significantly more complex, best seeds for comparing coding sequences are expected to depend not only on the sequence identity level, but also on the pattern of mutations, in particular transition-transversion biases. Intuitively, similar alignment models should produce similar behavior of seeds. Thus, one solution to seed proliferation is to group comparisons that exhibit similar behavior of seeds into *seed-equivalent *classes, such that all comparisons in one class are well served by the same optimal seed. Furthermore, it would be even more desirable to identify one or a small set of seeds suitable for a wide variety of evolutionary distances and mutation patterns, herein called *universal *seeds.

Our statistical regression analyses of seed sensitivities among four comparisons, chosen at various evolutionary distances, showed that for some pairs of comparisons seed behavior can be predicted closely with high-confidence. In particular, it was possible to identify two sets of models that have relatively different sensitivity ranges but very similar seed behavior, and therefore can be deemed seed-equivalent. Moreover, even at larger evolutionary distances more distant comparisons are predictive of closer ones. This conclusion is corroborated by several factors, including the length of the prediction interval, the pattern of outliers, and the ratio of predicted to optimal sensitivity (Table 1). For instance, at 2.5*σ *regression error, all but a few of the outliers perform better than predicted in the distant model compared to the closer one. In other words, distant comparisons contain more information than closer ones. One outstanding question is how to determine whether two models are close enough to be seed-equivalent. While we have not yet found a theoretical formulation, we investigated the relationship between alignment Markov models using a conventional distance measure between their probability distributions. The Kullback-Leibler Divergence (KLD) [23] can be applied on the space of alignment words X = {0, 1, *x*}^64 ^to produce a distance between the two models [see Additional file 3]:

$$KLD(P,Q)=\sum_{w\in X}p(w)\log\frac{p(w)}{q(w)}$$

*KLD*(*P, Q*) represents the relative entropy of *P *over *Q*, or the information gain about X when *P *is used instead of *Q*. The KLD measure is non-symmetrical, *i.e. KLD*(*P, Q*) ≠ *KLD*(*Q*, *P*), and therefore can capture unidirectional relationships. In Table 4, the more distant comparisons contain consistently more information than closer ones. (Note that we have judged comparisons based on the relative sequence similarity levels, for instance DOG (*p*_*d *_= 89.0%) is closer than MUS (*p*_*m *_= 85.0%).) The table also suggests that a possible cutoff for deciding seed-equivalence may be set between 1.0 and 2.0. Additional models and analyses will be needed, however, to validate and fine tune this criterion.

**Table 4:** Distances between models: KLD is the Kullback-Leibler Divergence.

**Comparisons**	*KLD *(*P, Q*)
CHK-DOG (DOG-CHK)	4.857 (4.077)
CHK-MUS (MUS-CHK)	2.351 (2.012)
CHK-ZFS (ZFS-CHK)	0.870 (0.885)
DOG-MUS (MUS-DOG)	0.468 (0.491)
DOG-ZFS (ZFS-DOG)	8.366 (9.997)
MUS-ZFS (ZFS-MUS)	5.352 (6.329)

Lastly, one natural question that arises is whether universal seeds exist for other types of comparisons, such as between genomic sequences. Our preliminary experiments using a {0, 1} Bernoulli model of alignment [7,19] and four different sequence similarity levels to represent different evolutionary distances (*p *= 0.65, 0.75, 0.85 and 0.95) indicate that, again, good seeds for the more distant comparisons will perform well on the closer ones [see Additional file 4]. Moreover, for weight 12, *p *= 0.65, 0.75, 0.85 form a seed-equivalent cluster and the optimal seed is shared among the four models, whereas for the larger weights (14, 16) the seed-equivalent groups are sparser and no one seed is optimal for all comparisons. These findings are consistent with the observations in [19]. Thus, although more analyses are needed to test it on various models, our simple strategy for selecting universal good seeds may be more widely applicable to a variety of sequence comparison problems.

## Conclusion

We performed a statistical analysis of seed sensitivities for four species-to-species cDNA-to-genome comparisons, spanning a wide range of evolutionary distances and mutation patterns, with the goal to determine criteria for selecting a small set of seeds that would perform well on a wide range of comparisons. In particular, grouping models that exhibit similar behavior of seeds into seed-equivalence classes could significantly reduce the number of optimal seeds. Most important for practical applications, the analyses showed that with high probability optimal and near-optimal seeds for the most distant available comparison will translate into good seeds for a wide range of comparisons. These insights, and the sets of optimal seeds predicted for the four comparisons and for a wide array of weights, represent a useful resource in guiding the selection of seeds for developing practical applications.

## Availability

Material referenced in the paper can be found in the Additional files below and at our website [24].

## Methods

### Optimal seeds

Given an alignment model, we calculate seed sensitivity in the {0, 1, x} model recursively as described in [11]. For convenience, we include a summary here.

Let M be a homogeneous order 2 Markov model of alignment, and $D(S)=\{Q,\Sigma ,A,q_{0},q_{a},q_{f}\}$ a deterministic finite automoton (DFA) that accepts all and only alignment strings that contain a seed match, built with the Aho-Corasick algorithm [25]. Here, Q denotes the set of states, Σ = {0, 1, *x*} is the alignment alphabet, A is the set of state transitions, and *q*_0_, *q*_*a*_, *q*_*f *_are the *start*, *accept *and *fail *states. We write $q\overset{b}{\underset{}{\to }}q^{\prime }$ if there is a DFA state transition from state *q *to state *q' *on the symbol *b *∈ Σ. The sensitivity of the seed *Sn*(*S*) then is the probability of all alignment words of length *L *= 64 accepted by the DFA. We calculate recursively the probabilities *P*(*q*, *t*, *α*), *q *∈ Q, *α *∈ Σ^3 ^that the automaton reaches state *q *after reading *t *input symbols ending in suffix *α *randomly generated from the alignment model. Then, the sensitivity of seed *S *is $Sn(S)=\sum_{\alpha \in \Sigma^{3}}P(q_{a},L,\alpha )$:

$$\begin{array}{lll} P(q,0,\varepsilon ) & = & \begin{array}{cc} 1, & \text{if }q=q_{0}\text{ and }0,\text{ otherwise} \end{array}\\ P(q,1,b_{1}) & = & \begin{array}{cc} P_{0}(b_{1})\sum_{q^{\prime }\overset{b_{1}}{\underset{}{\to q}}}P(q^{\prime },0,\varepsilon ), & \forall b_{1}\in \{0,1,x\} \end{array}\\ P(q,2,b_{1}b_{2}) & = & \begin{array}{cc} P_{1}(b_{2}|b_{1})\sum_{q^{\prime }\overset{b_{2}}{\underset{}{\to q}}}P(q^{\prime },1,b_{1}), & \forall b_{1},b_{2}\in \{0,1,x\} \end{array}\\ P(q,3,b_{1}b_{2}b_{3}) & = & \begin{array}{cc} P_{2}(b_{3}|b_{1}b_{2})\sum_{q^{\prime }\overset{b_{3}}{\underset{}{\to q}}}P(q^{\prime },2,b_{1}b_{2}), & \forall b_{1},b_{2},b_{3}\in \{0,1,x\} \end{array}\\ P(q,t,\delta b) & = & \begin{array}{cc} P_{2}(b|\delta )\sum_{q^{\prime }\overset{b}{\underset{}{\to q}}}\sum_{b_{0}\in \{0,1,x\}}P(q^{\prime },t-1,b_{0}\delta ), & \forall \delta \in {\left\{0,1,x\right\}}^{2} \end{array}, \end{array}$$

where *P*_0_, *P*_1 _and *P*_2 _are the marginal probabilities of the order 2 Markov model M, and ε is the empty word.

To determine optimal seeds, we use a hill-climbing heuristic [6,11], starting from a random seed and swapping any two distinct symbols with the goal to optimize the score locally. If a 'better' seed is found, it becomes the start seed for variations in the next cycle, until there are no changes to the optimal score. The procedure is applied 20 times for each weight *W *and for each combination (*n*_1_, *n*_*x*_, *n*_0_), where *n*_*i *_is the number of *i *symbols in the seed, satisfying *n*_1 _+ *n*_0 _+ *n*_*x *_= 22 and *n*_1 _+ 0.5·*n*_*x *_= *W*.

### Seed evaluation

We evaluate seed sensitivity both theoretically and empirically to identify good seeds for practical applications. To determine the effects of species divergence on the choice of seeds, we analyze comparisons between human and four other species (dog, mouse, chicken and zebrafish), which coarsely sample the range of vertebrate evolutionary distances.

#### Theoretical seed evaluation

Given a seed and an alignment model, the theoretical seed sensitivity can be calculated recursively as described above. To train the alignment models for the four sets of comparisons, exons in the human genome were determined from spliced genomic alignments of human mRNA and EST sequences, produced with the programs Sim4/ESTmapper [2,26]. A subset of coding exons and their reading frames were then determined by Fastx [27] matches against the SwissProt database [28]. Lastly, match statistics within the coding exon regions were collected from whole-genome alignments downloaded from the UCSC Genome Browser [29] and used to train the alignment models.

#### Empirical evaluation

This evaluation component tests the ability of a seed to accurately match dog, mouse, chicken and zebrafish coding exons with their orthologs in the human genome. For this purpose, reference sets of orthologous exons were constructed from homologous RefSeq gene pairs [30,31] as follows. Exon coordinates in the human mRNA and in the human genome were determined from Sim4 [26] alignments of the human mRNA sequence on the human genome HG17 produced with the high-throughput program ESTmapper [2]. These coordinates were then projected onto the mRNA of the other species via the pairwise mRNA-mRNA alignment. Starting with roughly 500 gene pairs for each comparison, this procedure produced 2,408 (dog), 4,198 (mouse), 4,869 (chicken) and 4,543 (zebrafish) exon pairs for use in our empirical analysis.

During the evaluation, dog, mouse, chicken and zebrafish coding sequences are searched against the human genome. The search program scans the input sequences, looking for seed matches of length 22 in the human genome. For efficiency, the search uses a bit-encoded index of 22 bp words (22-mers) in the genome, from which non-informative bits corresponding to 0 or *x *positions in the seed are removed. Empirical sensitivity is calculated as the fraction of human exons in the reference set that are detected by the seed: *Sn*^*e *^= *TP/*(*TP *+ *FN*) [32].

### Statistical analysis

We compare the distributions of seed sensitivities between any two comparisons (DOG, MUS, CHK and ZFS) using statistical regression methods. We used a *t*-test to determine the 95% confidence interval ($\hat{y}$ - *t*_*α*/2_*σ*_*y*_, $\hat{y}$ + *t*_*α*/2_*σ*_*y*_) for the projection $\hat{y}$ = *R*(*x*) of a sensitivity value *x *of a seed from the reference model to the compared model. Here, *α *= 0.05, *t*_*α*/2 _= 1.96, and *σ*_*y *_is the estimated regression standard error of prediction for a given *x *value. Because the number of values is large, *σ*_*y *_≃ *σ *for all *y*. A non-linear regression method with an order 3 polynomial was applied to the data: *R*(*x*) = (*a *+*bx*_max _+ $cx_{\max}^{2}$ + $dx_{\max}^{3}$). Lastly, outliers were determined as data points *x *whose values *y *in the compared model fall below or above the projected interval: *y *- $\hat{y}$ > 2.5*σ *or $\hat{y}$ - *y *> 2.5*σ*.

## Authors' contributions

LZ performed the analyses and contributed to the drafting of the manuscript. JS provided computational support and critically reviewed the manuscript. LF designed the experiments, directed the project, and drafted the article. All authors have read and approved the final version of the manuscript.

## Supplementary Material

Additional File 1 Scatterplots of seed sensitivity values between pairs of comparisons. This collection of figures shows the scatterplots of seed sensitivity values and the fitted regression curves between any two models in (DOG, MUS, CHK, ZFS). Click here for file

Additional File 2 Optimal seeds for the CHK, DOG, MUS and ZFS comparisons. This table lists the seeds optimized for the DOG, MUS, CHK and ZFS comparisons, for weights *W *= 10..16 and for all (*n*_1_, *n*_0_, *n*_*x*_) combinations, obtained using hill-climbing. For larger weights (*e.g*., *W *≥ 16), the fixed span *k *= 22 may significantly constrain the range of seeds, and therefore the seeds produced under this model may not be optimal in practice. Click here for file

Additional File 3 Efficient calculation of the KLD for two Markov models. This appendix describes a recursive procedure for calculating the Kullback-Leibler divergence between two Markov models efficiently. Click here for file

Additional File 4 Regression analysis of seed sensitivity distributions for genomic sequence comparison. This table contains the results from the statistical regression analysis of seed sensitivity distributions, similarly to Table 1, but for the case of genomic sequence comparisons. The four models are characterized by the sequence identity levels: *p *= 0.65, 0.75, 0.85, 0.95. Click here for file
