# Supplementary figures and images for: Universal seeds for cDNA-to-genome comparison
Source: BMC Bioinformatics. 2008 Jan 23;9:36. doi: 10.1186/1471-2105-9-36 (PMC2375135; doi:10.1186/1471-2105-9-36)

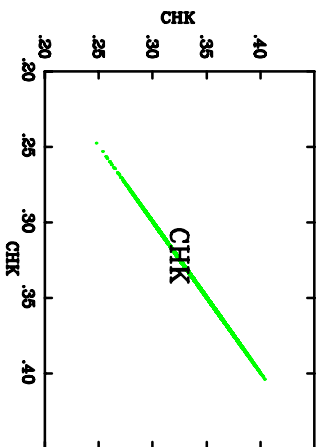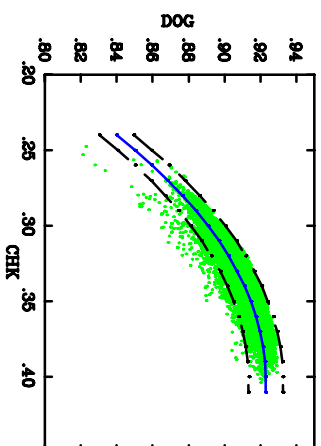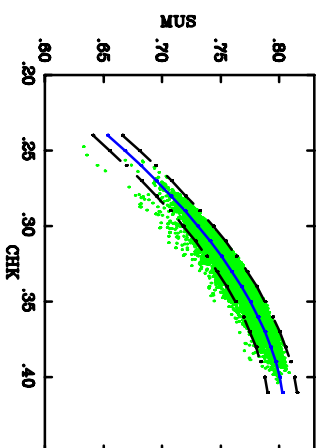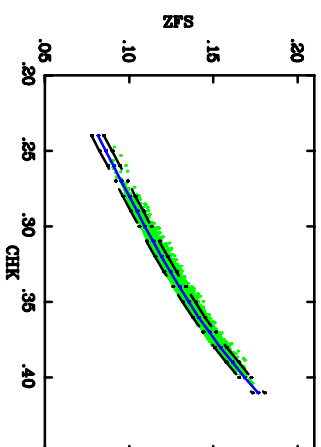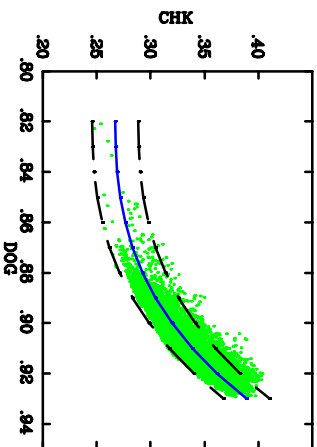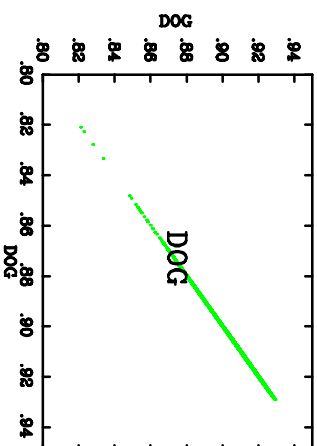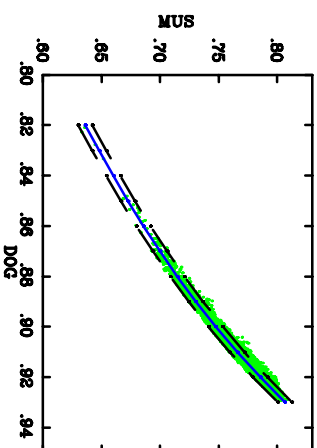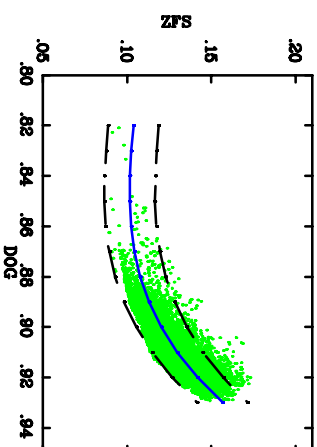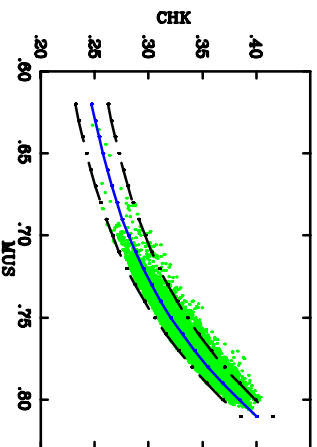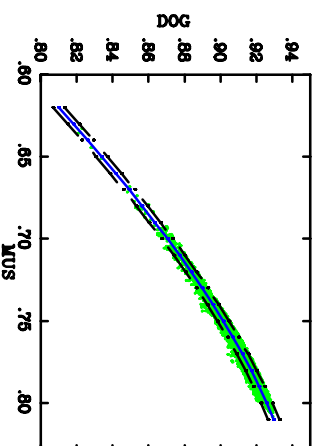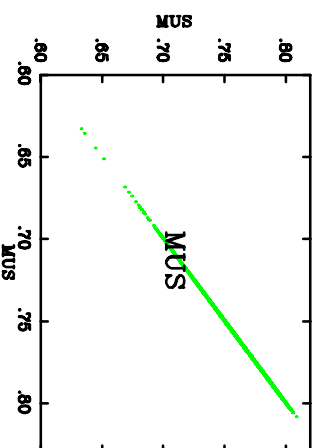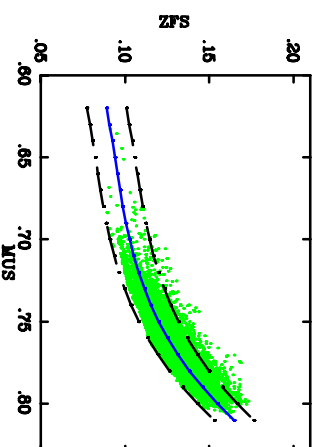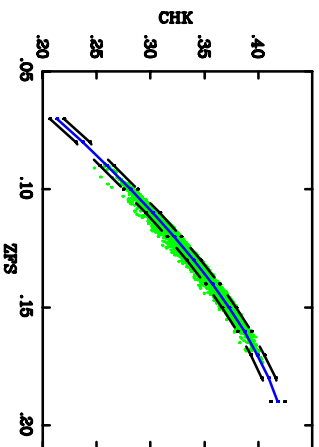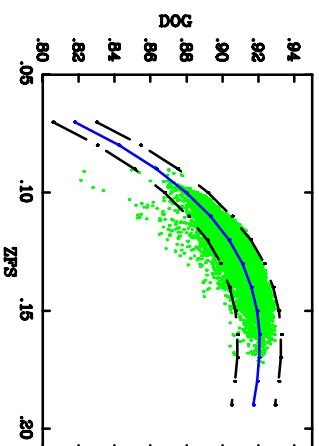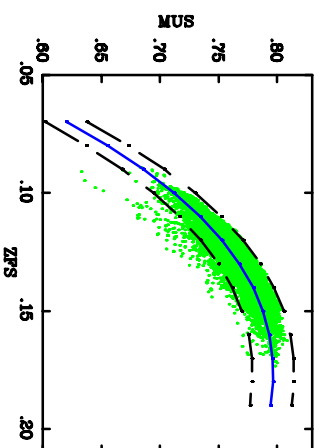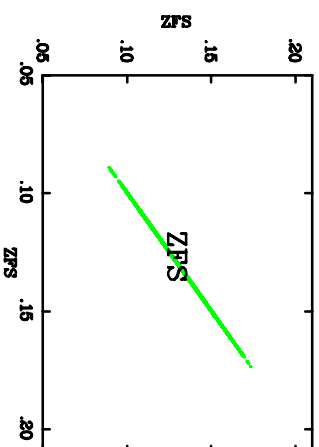

Supplement: Additional File 1 — Scatterplots of seed sensitivity values between pairs of comparisons. This collection of figures shows the scatterplots of seed sensitivity values and the fitted regression curves between any two models in (DOG, MUS, CHK, ZFS). [file 1471-2105-9-36-S1.pdf]
