# Supplementary material for: Universal seeds for cDNA-to-genome comparison
Source: BMC Bioinformatics. 2008 Jan 23;9:36. doi: 10.1186/1471-2105-9-36 (PMC2375135; doi:10.1186/1471-2105-9-36)
Supplement: Additional File 2 — Optimal seeds for the CHK, DOG, MUS and ZFS comparisons. This table lists the seeds optimized for the DOG, MUS, CHK and ZFS comparisons, for weights W = 10..16 and for all (n1, n0, nx) combinations, obtained using hill-climbing. For larger weights (e.g., W ≥ 16), the fixed span k = 22 may significantly constrain the range of seeds, and therefore the seeds produced under this model may not be optimal in practice. [file 1471-2105-9-36-S2.pdf]

Additional file 2. Seeds optimized for the CHK, DOG, MUS and ZFS comparisons, for weight W=10..16, using hill-climbing. For larger weights (*e.g.*, $W \geq 16$), the fixed span $k = 22$ may significantly constrain the range of seeds, and therefore the seeds produced under this model may not be optimal in practice.

Comparison	n_1	n_0	n_x	W	Order	Sensitivity	Seed
CHK	3	5	14	10	3	0.83728063	xxxx0x1x01x01x0x0xxxxx
CHK	4	6	12	10	3	0.85626162	xxxxx0110110xxxx0xxx00
CHK	5	7	10	10	3	0.87338128	xxxx0110110x1x0xxxx000
CHK	6	8	8	10	3	0.88738357	xxx10110110x1x0xxx0000
CHK	7	9	6	10	3	0.89794275	xxx1011011011xxx000000
CHK	8	10	4	10	3	0.90319464	x1x11011011xx100000000
CHK	9	11	2	10	3	0.90338191	1x11011011x11000000000
CHK	10	12	0	10	3	0.89136265	1011011011011100000000
DOG	3	5	14	10	3	0.99740595	xxx1x01xxx0x00xx0x1xxx
DOG	4	6	12	10	3	0.99810149	xxx1x0x0xx0x1x011xxx00
DOG	5	7	10	10	3	0.99855304	1xx110x1x0x00x0x1xxx00
DOG	6	8	8	10	3	0.99890714	1xx10xx0x1x011x1x00000
DOG	7	9	6	10	3	0.99908146	x1x110110xxx011x000000
DOG	8	10	4	10	3	0.99919518	11xx1101x011x100000000
DOG	9	11	2	10	3	0.99912045	11x11011x1110000000000
DOG	10	12	0	10	3	0.99866801	1111011011011000000000
MUS	3	5	14	10	3	0.98848711	xxx1x0xx00x0xxx10x1xxx
MUS	4	6	12	10	3	0.99091378	xxx1x0x0x10x1x01xxxx00
MUS	5	7	10	10	3	0.99244133	xxx110x1x0x00x0x1xx100
MUS	6	8	8	10	3	0.99359588	xxx1x010x1011xx1x00000
MUS	7	9	6	10	3	0.99434544	1xx10110x1x011xx000000
MUS	8	10	4	10	3	0.99463910	1xx1011011xx1100000000
MUS	9	11	2	10	3	0.99388665	1x110110110x1100000000
MUS	10	12	0	10	3	0.99178168	1111011011011000000000
ZFS	3	5	14	10	3	0.57999806	xxxxx0x0x10x10x1x0xxxx
ZFS	4	6	12	10	3	0.61019187	xxx0xxxx0110110xxxxx00
ZFS	5	7	10	10	3	0.64069130	xxxx0110110x1x0xxxx000
ZFS	6	8	8	10	3	0.66731487	xxx10110110x1xxxx00000
ZFS	7	9	6	10	3	0.69173419	xxx1011011011xxx000000
ZFS	8	10	4	10	3	0.70636903	x1x11011011xx100000000
ZFS	9	11	2	10	3	0.71139280	1x11011011x11000000000
ZFS	10	12	0	10	3	0.69896781	1101101101101100000000

Comparison	n_1	n_0	n_x	W	Order	Sensitivity	Seed
CHK	3	3	16	11	3	0.74195344	xxxxxxx01x0x1xx10xxxxx
CHK	4	4	14	11	3	0.76968148	xxxxxx0x1x010x10x1xxxx
CHK	5	5	12	11	3	0.79068444	xxx1x0x0x10x10x1x01xxx
CHK	6	6	10	11	3	0.80905262	xxx110x1x01x010x1xxx00
CHK	7	7	8	11	3	0.82391087	xxx10110110x1x01xxx000
CHK	8	8	6	11	3	0.83340361	xxx11011011011xxx00000
CHK	9	9	4	11	3	0.83735948	1xx1011011011xx1000000
CHK	10	10	2	11	3	0.83639338	x11011011011x110000000
CHK	11	11	0	11	3	0.82520282	1101101101101110000000
DOG	3	3	16	11	3	0.99247455	xxx1x0xxxx0xx0xxx11xxx
DOG	4	4	14	11	3	0.99444933	xxxx1x0xx0x0x1x01xx1xx
DOG	5	5	12	11	3	0.99575073	xx1x01xx10xx00x0x1xx1x
DOG	6	6	10	11	3	0.99659788	1xx1x0x0xx0x1x011x1x00
DOG	7	7	8	11	3	0.99719461	1xx110x1x0x0x1011xx000
DOG	8	8	6	11	3	0.99753679	1xx10110xxx011x1100000
DOG	9	9	4	11	3	0.99763545	11x110110x0x1x11000000
DOG	10	10	2	11	3	0.99731712	1x11011011x11100000000
DOG	11	11	0	11	3	0.99656179	1111011011011100000000
MUS	3	3	16	11	3	0.97219125	xxx1x0xxxx0xx01xxx1xxx
MUS	4	4	14	11	3	0.97810379	xxxx10x1x01x0x0xxx1xxx
MUS	5	5	12	11	3	0.98163810	xxxx1x01x0x0x1x011xxx0
MUS	6	6	10	11	3	0.98435221	xxx110x1x01x0x0x1xx100
MUS	7	7	8	11	3	0.98601574	1xx10xx010x1x011xx1000
MUS	8	8	6	11	3	0.98674741	1xx10110x1x011xx100000
MUS	9	9	4	11	3	0.98680584	11x1101x011xx110000000
MUS	10	10	2	11	3	0.98527708	110110110x011x11000000
MUS	11	11	0	11	3	0.98123899	1110110110111100000000
ZFS	3	3	16	11	3	0.45148616	xxxxxx0x10x1xx10xxxxxx
ZFS	4	4	14	11	3	0.48468875	xxxxxxx0110x10x1x0xxxx
ZFS	5	5	12	11	3	0.51332571	xxxx10x1x010x10x1xxxx0
ZFS	6	6	10	11	3	0.53946318	xxxx10110110x1x0xxxx00
ZFS	7	7	8	11	3	0.56376093	xxx10110110x1x01xxx000
ZFS	8	8	6	11	3	0.58517406	xxx11011011011xxx00000
ZFS	9	9	4	11	3	0.59562187	1xx1011011011xx1000000
ZFS	10	10	2	11	3	0.60418946	11x11011011x1100000000
ZFS	11	11	0	11	3	0.58810764	1101101101101110000000

Comparison	n_1	n_0	n_x	W	Order	Sensitivity	Seed
CHK	3	1	18	12	3	0.60384548	xxxx1xx1xx1x0xxxxxxxxx
CHK	4	2	16	12	3	0.66164144	xxx1xx10x1xx1x0xxxxxxx
CHK	5	3	14	12	3	0.69437357	xxxxx1x0110x1xx10xxxxx
CHK	6	4	12	12	3	0.71760825	xxx1xx10x1x01x010x1xxx
CHK	7	5	10	12	3	0.73345458	x1xx110x1x010x10x1xxx0
CHK	8	6	8	12	3	0.74784826	1xx110x1x010x1011xxx00
CHK	9	7	6	12	3	0.75587173	x1x1101101x011xx1x0000
CHK	10	8	4	12	3	0.76175538	1xx1011011011xx1100000
CHK	11	9	2	12	3	0.75579292	11x11011011x1110000000
CHK	12	10	0	12	3	0.74171904	1101100011011011011000
DOG	3	1	18	12	3	0.97642269	xxxx1xx1xxxx0xxx1xxxxx
DOG	4	2	16	12	3	0.98527755	xxxxxxx01xx1x0x1xx1xxx
DOG	5	3	14	12	3	0.98918094	xxx110xxxx0xx01xx11xxx
DOG	6	4	12	12	3	0.99152865	x1xx110xx0x0x1x01xx1xx
DOG	7	5	10	12	3	0.99285630	1xx110x1x0x0xx011x1xx0
DOG	8	6	8	12	3	0.99380312	1xx110x1x010xx011x1x00
DOG	9	7	6	12	3	0.99429562	1x1101x0x0x1x011x11000
DOG	10	8	4	12	3	0.99432140	11x110x1011x1x11000000
DOG	11	9	2	12	3	0.99346971	11x11010110011x1100000
DOG	12	10	0	12	3	0.99167340	1111101101101110000000
MUS	3	1	18	12	3	0.93072527	xxxxxxxxx0x1xx1xx1xxxx
MUS	4	2	16	12	3	0.95185995	xxxxxxx01xx1x0x1xx1xxx
MUS	5	3	14	12	3	0.96100882	xxx1x0xxxx0x1011xx1xxx
MUS	6	4	12	12	3	0.96730094	x1xx1x01x0x0x1x011xxxx
MUS	7	5	10	12	3	0.97052561	1xx1x01x0x0x1x011xx1x0
MUS	8	6	8	12	3	0.97298419	1xx110x1x010xx011x1x00
MUS	9	7	6	12	3	0.97373714	1x1101x010x1x011xx1000
MUS	10	8	4	12	3	0.97293383	11x11011011xxx11000000
MUS	11	9	2	12	3	0.96980689	111x11011011x110000000
MUS	12	10	0	12	3	0.96371756	1110110110110111000000
ZFS	3	1	18	12	3	0.31417791	xxxxxxxxx10x1xx1xxxxxx
ZFS	4	2	16	12	3	0.36447368	xxx1xx10x1xx1x0xxxxxxx
ZFS	5	3	14	12	3	0.39733897	xxxxx1x0110x1xx10xxxxx
ZFS	6	4	12	12	3	0.42268099	xxx1xx10x1x01x010x1xxx
ZFS	7	5	10	12	3	0.44331033	xxx10x1x110110x1xxxx00
ZFS	8	6	8	12	3	0.46281008	xxx1011x110110x1xxx000
ZFS	9	7	6	12	3	0.47741461	xxxx11011011011xx10000
ZFS	10	8	4	12	3	0.48940267	x1x11011011011xx100000
ZFS	11	9	2	12	3	0.48706348	11x110x101101101100000
ZFS	12	10	0	12	3	0.47606922	1101100011011011011000

Comparison	n_1	n_0	n_x	W	Order	Sensitivity	Seed
CHK	4	0	18	13	3	0.46258586	xxxxxx1xx1xxxxx1xx1xxx
CHK	5	1	16	13	3	0.56459220	xxxxxx1xx10x1xx1xx1xxx
CHK	6	2	14	13	3	0.61392990	xxx1xx10x1xx1x01xx1xxx
CHK	7	3	12	13	3	0.63815207	xxx1xx10110x1x01xx1xxx
CHK	8	4	10	13	3	0.65492852	xxx10x1x01101101xx1xxx
CHK	9	5	8	13	3	0.66670994	1xx110x1xx1011011xxx00
CHK	10	6	6	13	3	0.67556941	1xx1011x1x011011xx1000
CHK	11	7	4	13	3	0.67815337	11x1101101x011xx110000
CHK	12	8	2	13	3	0.67083313	11x110x011011011011000
CHK	13	9	0	13	3	0.65340875	1101101101101110110000
DOG	4	0	18	13	3	0.93337760	xxx1xx1xxxxx1xx1xxxxxx
DOG	5	1	16	13	3	0.97059458	x1xx1xx1x0xxx1xx1xxxxx
DOG	6	2	14	13	3	0.98005107	x1xx1xx1x0xxx10x1xx1xx
DOG	7	3	12	13	3	0.98407358	xxx11x110xx0xxx1011xxx
DOG	8	4	10	13	3	0.98667435	1xx110x1xx100x0x1x1xx1
DOG	9	5	8	13	3	0.98777783	x11x110x1x0x0x1011x1x0
DOG	10	6	6	13	3	0.98829163	11x1101x1x0xx011x11000
DOG	11	7	4	13	3	0.98811695	111x110xx011011x110000
DOG	12	8	2	13	3	0.98686346	11x11100101101 1x110000
DOG	13	9	0	13	3	0.98365690	1111011011011101100000
MUS	4	0	18	13	3	0.84917666	xxx1xx1xxxxx1xx1xxxxxx
MUS	5	1	16	13	3	0.91560775	xxxxx1xx1x0x1xx1xx1xxx
MUS	6	2	14	13	3	0.93658186	xxx1xx1x0x1xx101xx1xxx
MUS	7	3	12	13	3	0.94511433	1xx110x1xx1x0x0xxx1xx1
MUS	8	4	10	13	3	0.95055729	1xx1x1x0x001xx1x011xx1
MUS	9	5	8	13	3	0.95336367	1xx110x1x01xx1011x1x00
MUS	10	6	6	13	3	0.95380719	1x1101xx10x1x011x11000
MUS	11	7	4	13	3	0.95135444	11x110x011011011xx1000
MUS	12	8	2	13	3	0.94700788	11x1100011011011x11000
MUS	13	9	0	13	3	0.93704606	1111011011011101100000
ZFS	4	0	18	13	3	0.21200181	xxxxxx1xx1xxxxx1xx1xxx
ZFS	5	1	16	13	3	0.27925522	xxxxxx1xx10x1xx1xx1xxx
ZFS	6	2	14	13	3	0.31704925	xxx1xx1x01xx10x1xx1xxx
ZFS	7	3	12	13	3	0.34006938	xxxxx1x011011xx10x1xxx
ZFS	8	4	10	13	3	0.35887580	xxx10x1x01101101xx1xxx
ZFS	9	5	8	13	3	0.37306891	xxx1011x11011011xxxx00
ZFS	10	6	6	13	3	0.38534432	xxx1011x11011011xx1000
ZFS	11	7	4	13	3	0.39227292	11x11011011011xxx10000
ZFS	12	8	2	13	3	0.39728765	11x11011011011x1100000
ZFS	13	9	0	13	3	0.38148832	1110110110110110110000

Comparison	n_1	n_0	n_x	W	Order	Sensitivity	Seed
CHK	6	0	16	14	3	0.43875936	xxx1xx1xx1xxxxx1xx1xx1
CHK	7	1	14	14	3	0.51745751	xxx1xx1xx1011xx1xx1xxx
CHK	8	2	12	14	3	0.55423555	xxx1xx101xx1x011xx1xx1
CHK	9	3	10	14	3	0.57319177	xxx1x1101101xx110x1xxx
CHK	10	4	8	14	3	0.58383918	1xx110110110xxx1011xxx
CHK	11	5	6	14	3	0.58937866	1xx1x110110x1x011x1100
CHK	12	6	4	14	3	0.59072013	11x11011011011xxx11000
CHK	13	7	2	14	3	0.58289040	11x1101011011011x11000
CHK	14	8	0	14	3	0.56738155	1101100011011011011011
DOG	6	0	16	14	3	0.92826749	xxx1xx1x1xxx1xx1xx1xxx
DOG	7	1	14	14	3	0.96259713	x1xx1xx1x0xxx1xx1x1xx1
DOG	8	2	12	14	3	0.97237081	1xx1x1xx10xxx1x011xx1x
DOG	9	3	10	14	3	0.97640590	xx11x1101xxx0x1x011x1x
DOG	10	4	8	14	3	0.97843995	11x110x1x0x0xx011x1x11
DOG	11	5	6	14	3	0.97906895	11xx11011x0x0x1011x110
DOG	12	6	4	14	3	0.97816304	111x110x0x1011011x1100
DOG	13	7	2	14	3	0.97595143	111x1100101101 1x111000
DOG	14	8	0	14	3	0.97100308	1111011011011101110000
MUS	6	0	16	14	3	0.83717923	1xx1xx1xxxxx1xx1xx1xxx
MUS	7	1	14	14	3	0.89454118	xxx1x1xx1x011xx1xx1xxx
MUS	8	2	12	14	3	0.91398282	1xx1x1x01xx110x1xx1xxx
MUS	9	3	10	14	3	0.92258435	1xx1x1x010x1xx1x011xx1
MUS	10	4	8	14	3	0.92505391	11x110x1x0x0x1011x1xx1
MUS	11	5	6	14	3	0.92608633	11xx110x1x010x1011x110
MUS	12	6	4	14	3	0.92217353	11x11010x1011x011x1100
MUS	13	7	2	14	3	0.91428209	11x110110110x011011100
MUS	14	8	0	14	3	0.90068032	1111011010011011101100
ZFS	6	0	16	14	3	0.19287589	xxx1xx1xx1xxxxx1xx1xx1
ZFS	7	1	14	14	3	0.24032437	xxx1xx1xx1011xx1xx1xxx
ZFS	8	2	12	14	3	0.26771068	xxx1xx1011x110x1xx1xxx
ZFS	9	3	10	14	3	0.28419633	xxx1xx1011011x110x1xxx
ZFS	10	4	8	14	3	0.29677403	xxxx11011011x11011xxx0
ZFS	11	5	6	14	3	0.30513299	1xx110x1x11011011xx100
ZFS	12	6	4	14	3	0.30907954	1xx110110x1011011x1100
ZFS	13	7	2	14	3	0.30776080	1x11011011011011x11000
ZFS	14	8	0	14	3	0.29942008	1101100011011011011011

Comparison	n_1	n_0	n_x	W	Order	Sensitivity	Seed
CHK	8	0	14	15	3	0.40379583	1xx1xx1x1xx1xxx1xx1xx1
CHK	9	1	12	15	3	0.46463026	1xx1xx1x1x011xx1xx1xx1
CHK	10	2	10	15	3	0.49140552	1xx1x1101xx1x011xx1xx1
CHK	11	3	8	15	3	0.50417856	1xx1x1101xx1x011011xx1
CHK	12	4	6	15	3	0.50963939	11x110xxx1011011011xx1
CHK	13	5	4	15	3	0.50865232	11x1101101101x011xx110
CHK	14	6	2	15	3	0.49968149	1x110110110110111x1100
CHK	15	7	0	15	3	0.48412273	1101110011011011011011
DOG	8	0	14	15	3	0.92000589	1xx1xx1x1xx1xxx1xx1xx1
DOG	9	1	12	15	3	0.95174388	1xx1x1xx1x0x1x11xx1xx1
DOG	10	2	10	15	3	0.96118772	1x11x1x0xxx10x1xx11x11
DOG	11	3	8	15	3	0.96492446	1x11x1101xxx0x1x011x11
DOG	12	4	6	15	3	0.96521973	11x110x1x010x1011x1x11
DOG	13	5	4	15	3	0.96357640	11x110110110x0x111x110
DOG	14	6	2	15	3	0.95981547	11x11101101001 1x111100
DOG	15	7	0	15	3	0.95350303	1111011011011101111000
MUS	8	0	14	15	3	0.81809643	1xx1xx1x1xx1xxx1xx1xx1
MUS	9	1	12	15	3	0.86772995	1xx1x1xx1x011xx1xx1xx1
MUS	10	2	10	15	3	0.88344319	1xx1xx1101xxx011x11xx1
MUS	11	3	8	15	3	0.89014305	11x110x1x01xx1011x1xx1
MUS	12	4	6	15	3	0.88963068	11x110x1x010x1011x1x11
MUS	13	5	4	15	3	0.88397230	11x1110x1x01011011x110
MUS	14	6	2	15	3	0.87368184	11x1100011011011011x11
MUS	15	7	0	15	3	0.85766920	1111011011011011101100
ZFS	8	0	14	15	3	0.16693400	1xx1xx1xx1xxxx11xx1xx1
ZFS	9	1	12	15	3	0.20103797	xxx1xx1x11011xx1xx1xx1
ZFS	10	2	10	15	3	0.22013021	xxx1x11011x11011xx1xxx
ZFS	11	3	8	15	3	0.23137427	xxx1x1101101xx11011xx1
ZFS	12	4	6	15	3	0.23738828	1xx101101xx11011011xx1
ZFS	13	5	4	15	3	0.24199948	11x1101101xx11011x1100
ZFS	14	6	2	15	3	0.24133046	11x11011011011011x1100
ZFS	15	7	0	15	3	0.23202790	1101101101101101110110

Comparison	n_1	n_0	n_x	W	Order	Sensitivity	Seed
CHK	10	0	12	16	3	0.36119259	1xx11x1xx1x1xx1xx11xx1
CHK	11	1	10	16	3	0.40667331	1xx1xx1x1101xx11x11xx1
CHK	12	2	8	16	3	0.42623965	1xx1x1101xx11011x11xx1
CHK	13	3	6	16	3	0.43097172	11x1x1101101xx11011xx1
CHK	14	4	4	16	3	0.43282481	11x110xx11011011011x11
CHK	15	5	2	16	3	0.42365228	11x11011011011011 1x110
CHK	16	6	0	16	3	0.40518335	1110110110110110110111
DOG	10	0	12	16	3	0.90756041	1xx11x1xx1x1xx1xx11xx1
DOG	11	1	10	16	3	0.93641983	x1x11x1xxx011x1xx11x11
DOG	12	2	8	16	3	0.94489121	11x11xx1101xxx011x11x1
DOG	13	3	6	16	3	0.94611468	11x110x1x01xx1011x1111
DOG	14	4	4	16	3	0.94424328	111x110x1x01011011x111
DOG	15	5	2	16	3	0.93852120	11x1101101001110111x11
DOG	16	6	0	16	3	0.93009212	1101111010011011011111
MUS	10	0	12	16	3	0.79029836	1xx11x1xx1x1xx1xx11xx1
MUS	11	1	10	16	3	0.83159389	1xx1x1xx11011x1xx11xx1
MUS	12	2	8	16	3	0.84412830	11x1x1x01xx110x1x11x11
MUS	13	3	6	16	3	0.84458272	11x1101xxx011x11011x11
MUS	14	4	4	16	3	0.83894512	11x110110x011x11011x11
MUS	15	5	2	16	3	0.82616275	111x11011001011011x111
MUS	16	6	0	16	3	0.80831815	1110111011011011011110
ZFS	10	0	12	16	3	0.13945649	1xx1xx1x1xx1xx11x11xx1
ZFS	11	1	10	16	3	0.16349428	1xx1xx1x11011x11xx1xx1
ZFS	12	2	8	16	3	0.17668242	1xx1x11011x11011xx1xx1
ZFS	13	3	6	16	3	0.18350131	1xx1x11011011x11011xx1
ZFS	14	4	4	16	3	0.18630980	11xx11011011x11011x110
ZFS	15	5	2	16	3	0.18426480	11x11011011011011 1x110
ZFS	16	6	0	16	3	0.17636838	1101111011011011011011
