# Supplementary material for: Universal seeds for cDNA-to-genome comparison
Source: BMC Bioinformatics. 2008 Jan 23;9:36. doi: 10.1186/1471-2105-9-36 (PMC2375135; doi:10.1186/1471-2105-9-36)
Supplement: Additional File 3 — Efficient calculation of the KLD for two Markov models. This appendix describes a recursive procedure for calculating the Kullback-Leibler divergence between two Markov models efficiently. [file 1471-2105-9-36-S3.pdf]

**Additional file 3. Efficient calculation of the Kullback-Leibler divergence (KLD) for two Markov models**

Let $\mathcal{M}_1$ and $\mathcal{M}_2$ be two Markov models of order $k = 3$, characterized by the probability distributions $P$ and $Q$ on the space of alignment words $\mathcal{X} = \{0, 1, x\}^L$. To compute $KLD(P, Q)$, we divide the contributions from words $w$ in groups based on the last 3 digits in the word, and calculate the values recursively for increasing word lengths $|w| = m \leq L$:

$$KLD(P, Q) = \sum_{a_1 a_2 a_3 \in \{0,1,x\}^3} KLD^{(L)}(P, Q; a_1 a_2 a_3) \tag{1}$$

Let $\lambda \in \{0, 1, x\}^{m-3}, b \in \{0, 1, x\}$, for $m \geq 3$.

$$\begin{aligned}
& KLD^{(m+1)}(P, Q; a_1 a_2 a_3) \qquad (2)\\
= & \sum_\lambda \sum_b p(\lambda b a_1 a_2 a_3) \log \frac{p(\lambda b a_1 a_2 a_3)}{q(\lambda b a_1 a_2 a_3)} \\
= & \sum_\lambda \sum_b p(\lambda b a_1 a_2) \cdot \mu_P(a_3 | b a_1 a_2) \Big( \log \frac{p(\lambda b a_1 a_2)}{q(\lambda b a_1 a_2)} + \log \frac{\mu_P(a_3 | b a_1 a_2)}{\mu_Q(a_3 | b a_1 a_2)} \Big) \\
= & \sum_\lambda \sum_b p(\lambda b a_1 a_2) \mu_P(a_3 | b a_1 a_2) \log \frac{p(\lambda b a_1 a_2)}{q(\lambda b a_1 a_2)} \\
+ & \sum_\lambda \sum_b p(\lambda b a_1 a_2) \mu_P(a_3 | b a_1 a_2) \log \frac{\mu_P(a_3 | b a_1 a_2)}{\mu_Q(a_3 | b a_1 a_2)} \\
= & \sum_b \mu_P(a_3 | b a_1 a_2) \cdot \Big[ \sum_\lambda p(\lambda b a_1 a_2) \log \frac{p(\lambda b a_1 a_2)}{q(\lambda b a_1 a_2)} \Big] + \sum_b K(a_3; b a_1 a_2) \cdot \sum_\lambda p(\lambda b a_1 a_2) \\
= & \sum_b \mu_P(a_3 | b a_1 a_2) \cdot KLD^{(m)}(P, Q; b a_1 a_2) + \sum_b K(a_3; b a_1 a_2) \cdot P^{(m)}(b a_1 a_2)
\end{aligned}$$

where

$$K(a_3; b a_1 a_2) = \mu_P(a_3 | b a_1 a_2) \log \frac{\mu_P(a_3 | b a_1 a_2)}{\mu_Q(a_3 | b a_1 a_2)} \tag{3}$$

is a constant, and

$$P^{(m)}(b a_1 a_2) = \sum_\lambda p(\lambda b a_1 a_2) \tag{4}$$

Note that $P^{(m)}(b a_1 a_2)$ can be calculated *a priori* with the recurrences:

$$
\begin{aligned}
P^{(m+1)}(a_1a_2a_3) &= \sum_{\lambda b} p(\lambda b a_1 a_2 a_3) \\
&= \sum_{\lambda} \sum_{b} p(\lambda b a_1 a_2) \mu_P(a_3|ba_1a_2) \\
&= \sum_{b} \mu_P(a_3|ba_1a_2) (\sum_{\lambda} p(\lambda b a_1 a_2)) \\
&= \sum_{b} \mu_P(a_3|ba_1a_2) P^{(m)}(ba_1a_2)
\end{aligned} \tag{5}
$$

For $L = 64$, these recurrences will generate $64 \times 3^3$ intermediate values, which are later used in the calculation of $KLD^{(m)}$. Hence, the KLD distance can be computed effciently in $\mathcal{O}(L)$ time.
