# Supplementary material for: Universal seeds for cDNA-to-genome comparison
Source: BMC Bioinformatics. 2008 Jan 23;9:36. doi: 10.1186/1471-2105-9-36 (PMC2375135; doi:10.1186/1471-2105-9-36)
Supplement: Additional File 4 — Regression analysis of seed sensitivity distributions for genomic sequence comparison. This table contains the results from the statistical regression analysis of seed sensitivity distributions, similarly to Table 1, but for the case of genomic sequence comparisons. The four models are characterized by the sequence identity levels: p = 0.65, 0.75, 0.85, 0.95. [file 1471-2105-9-36-S4.pdf]

Additional file 4: Seed sensitivity distributions between models for the case of genomic sequence comparisons. $S65, S75, S85, S95$ indicate sequence similarity levels: $p = 0.65, 0.75, 0.85, 0.95$. b_0, b_1, b_2 are the second order regression parameters, σ is standard deviation.

**Comp.**	**Seed**	$x_{\max}$	$y_{\max}$	b_0	b_1	b_2	σ	$T(x, y)$
W=12								
S95-S75	(12,10,0)	1.000	0.587	-295.556	296.136	0.000	0.006	0.953
S75-S95	(12,10,0)	0.587	1.000	0.988	0.042	-0.036	0.000	1.000
S85-S75	(12,10,0)	0.952	0.587	3.953	-9.210	5.958	0.001	0.993
S75-S85	(12,10,0)	0.587	0.952	0.315	1.739	-1.111	0.001	0.999
S65-S75	(12,10,0)	0.184	0.587	0.004	3.820	-3.413	0.002	1.000
S75-S65	(12,10,0)	0.587	0.184	-0.023	0.330	0.034	0.001	0.983
S85-S95	(12,10,0)	0.952	1.000	0.916	0.176	-0.092	0.000	1.000
S95-S85	(12,10,0)	1.000	0.952	-161.572	162.523	0.000	0.003	0.988
S65-S95	(12,10,0)	0.184	1.000	0.992	0.093	-0.257	0.000	1.000
S95-S65	(12,10,0)	1.000	0.184	-107.296	107.475	0.000	0.002	0.932
S65-S85	(12,10,0)	0.184	0.952	0.424	4.700	-9.883	0.001	0.999
S85-S65	(12,10,0)	0.952	0.184	1.400	-3.339	2.162	0.001	0.975
W=14								
S95-S75	(14,8,0)	1.000	0.388	7098.070	-14255.870	7158.200	0.004	0.970
S75-S95	(14,8,0)	0.388	1.000	0.964	0.180	-0.227	0.000	1.000
S85-S75	(14,8,0)	0.872	0.388	0.384	-0.997	1.148	0.001	0.995
S75-S85	(14,8,0)	0.388	0.872	0.255	2.208	-1.598	0.001	0.997
S65-S75	(14,8,0)	0.082	0.388	0.099	2.192	16.372	0.001	0.993
S75-S65	(14,8,0)	0.388	0.082	-0.028	0.368	-0.218	0.000	0.992
S85-S95	(14,8,0)	0.872	1.000	0.875	0.279	-0.156	0.000	1.000
S95-S85	(14,8,0)	1.000	0.872	6838.380	-13739.720	6902.220	0.003	0.982
S65-S95	(14,8,0)	0.082	1.000	0.969	0.721	-4.230	0.000	1.000
S95-S65	(14,8,0)	1.000	0.082	1426.951	-2866.418	1439.551	0.001	0.963
S65-S85	(14,8,0)	0.082	0.872	0.383	7.219	-15.135	0.002	0.994
S85-S65	(14,8,0)	0.872	0.082	-0.026	0.045	0.090	0.000	0.988
W=16								
S95-S75	(16,6,0	0.998	0.237	413.747	-839.888	426.397	0.003	0.974
S75-S95	(16,6,0	0.237	0.998	0.912	0.691	-1.388	0.000	0.999
S85-S75	(16,6,0)	0.751	0.237	0.032	-0.007	0.373	0.001	0.996
S75-S85	(16,6,0)	0.237	0.751	0.165	3.162	-2.926	0.002	0.994
S65-S75	(16,6,0)	0.035	0.237	0.062	2.400	74.000	0.001	0.989
S75-S65	(16,6,0)	0.237	0.035	-0.009	0.240	-0.234	0.000	0.995
S85-S95	(16,6,0)	0.751	0.998	0.808	0.482	-0.305	0.000	0.999
S95-S85	(16,6,0)	0.998	0.751	715.145	-1453.142	738.783	0.004	0.986
S65-S95	(16,6,0)	0.035	0.998	0.916	4.380	-58.000	0.000	0.999
S95-S65	(16,6,0)	0.998	0.035	52.896	-107.474	54.615	0.000	0.972
S65-S85	(16,6,0)	0.035	0.751	0.275	13.486	0.695	0.003	0.988
S85-S65	(16,6,0)	0.751	0.035	-0.019	0.073	-0.001	0.000	0.993
